# Telomere Dysfunction in Chronic Lymphocytic Leukemia

**DOI:** 10.3389/fonc.2020.612665

**Published:** 2021-01-15

**Authors:** Billy Michael Chelliah Jebaraj, Stephan Stilgenbauer

**Affiliations:** ^1^ Department of Internal Medicine III, University of Ulm, Ulm, Germany; ^2^ Klinik für Innere Medizin I, Universitätsklinikum des Saarlandes, Homburg, Germany

**Keywords:** chronic lymphocytic leukemia, telomere dysfunction, telomerase activation, genomic complexity, prognostic factor, clonal evolution

## Abstract

Telomeres are nucleprotein structures that cap the chromosomal ends, conferring genomic stability. Alterations in telomere maintenance and function are associated with tumorigenesis. In chronic lymphocytic leukemia (CLL), telomere length is an independent prognostic factor and short telomeres are associated with adverse outcome. Though telomere length associations have been suggested to be only a passive reflection of the cell’s replication history, here, based on published findings, we suggest a more dynamic role of telomere dysfunction in shaping the disease course. Different members of the shelterin complex, which form the telomere structure have deregulated expression and *POT1* is recurrently mutated in about 3.5% of CLL. In addition, cases with short telomeres have higher telomerase (TERT) expression and activity. TERT activation and shelterin deregulation thus may be pivotal in maintaining the minimal telomere length necessary to sustain survival and proliferation of CLL cells. On the other hand, activation of DNA damage response and repair signaling at dysfunctional telomeres coupled with checkpoint deregulation, leads to terminal fusions and genomic complexity. In summary, multiple components of the telomere system are affected and they play an important role in CLL pathogenesis, progression, and clonal evolution. However, processes leading to shelterin deregulation as well as cell intrinsic and microenvironmental factors underlying TERT activation are poorly understood. The present review comprehensively summarizes the complex interplay of telomere dysfunction in CLL and underline the mechanisms that are yet to be deciphered.

## Introduction

Telomeres are repetitive DNA sequences at the ends of the chromosomes that play a pivotal role in maintaining genomic stability by capping and protecting the ends from degradation and fusions. Maintenance of telomere length is a key for immortalization in cancers. In chronic lymphocytic leukemia (CLL), telomere length has been identified as an independent prognostic factor in various studies. In addition, the deregulation of different telomere components has a profound influence on the CLL pathomechanisms. The present review is thus aimed at summarizing the clinical and biological aspects of telomere shortening, mutations and deregulated expression of telomere associated genes, and mechanisms that are important for telomerase activation in CLL, to pave way for a deeper understanding of telomere dysfunction in CLL pathogenesis.

## Telomeres—Structure and Function

All eukaryotic chromosomes have specialized nucleo-protein structures called telomeres which cap the ends. The nucleic acid component of the telomeres comprises of long tracts of DNA repeat sequences, ending with a 3’ single stranded DNA overhang. In mammals, the telomere sequences consist of TTAGGG hexamers, repeated many kilo bases in length (1). In somatic cells, a part of the DNA sequence is lost at the ends of the chromosomes during each cell division due to the end-replication problem (2, 3). The telomeres at the chromosomal ends thus serve as buffer preventing loss of vital genetic information. The telomeric repeats are associated with a six-subunit protein complex called shelterin consisting of TRF1, TRF2, TIN2, TPP1, POT1, and RAP1. TRF1 and TRF2 bind directly to the double stranded telomere sequence and POT1 binds to the 3’ single stranded overhang. TIN2 and TPP1 link TRF1 and TRF2 and POT1 while RAP1 binds solely to TRF2 (4). The 3’ telomere overhang at the chromosomal ends loops to form the T-loop by strand invasion. The T-loop structure along with the shelterin prevents the chromosomal ends from being recognized as DNA damage, conferring genomic stability (5).

In stem cells, germ cells, and in various cancers, the telomere length is maintained, most commonly by the reverse transcriptase enzyme, telomerase (TERT). It is an RNA-dependent DNA polymerase that uses the telomerase RNA component (TERC) as a template to synthesize the telomeric DNA (1). Thus in somatic cells that lack telomerase expression, telomere shortening beyond a critical length activate the senescence checkpoints, beyond which the cells cannot proliferate in the absence of an active telomere length maintenance mechanism. Activation of telomerase is considered as one of the hallmarks of malignant transformation (6). In addition, certain neoplasms undergo telomerase-independent alternative lengthening of telomeres (ALT), a recombination dependent pathway that utilizes telomeres of adjacent chromosomes as template for elongation and maintenance of critical telomere length (7, 8). In CLL, deregulations of various components of the telomere machinery such as length of telomeres, telomerase, and shelterin expression, and recurrent, activating *POT1* mutations point to a global telomere dysfunction that plays an important role in disease pathogenesis and evolution.

## Telomere Dysfunction and Tumorigenesis

The primary role of telomeres is to confer genomic stability. The shelterin complex shields the telomeres from activation of the DNA damage response signaling at the telomeres. In particular, TRF2 of the sheltein complex is important to prevent activation of the ATM (9) and subsequently non-homologous end joining (NHEJ) (10, 11) while POT1 suppresses ATR signaling (12) activation at the telomeres. Critical telomere shortening leads to uncapping of the ends and activation of senescence checkpoints. This is an important tumor suppressor mechanism that functions to eliminate potentially harmful, pre-malignant clones.

Progressive shortening of telomeres by knocking out *Terc* and crossing through generations G1 to G6 by knocking out *Terc* led to increased incidences of spontaneous malignancies and decreased stress response and survival (13). Dysfunctional telomeres lead to intra or inter chromosomal end fusions resulting in the formation of dicentric chromosomes that undergo breakage at the anaphase. This phenomenon is known as breakage-fusion-bridge (BFB) cycle which leads to genomic complexity. Evidences of such BFB events were found in many different cancer types (14, 15). Using murine models it was further demonstrated that loss of checkpoint genes such as *TP53* along with telomere dysfunction led to development of cancers due to non-reciprocal translocations caused by BFB events (16).

Of note, length of telomeres within a cell substantially varies between the different chromosomes and it was identified that the presence of one or more critically short telomeres and not the average telomere length dictates cellular senescence versus proliferation (17). Though the activation of telomerase or ALT mediated telomere maintenance is important for cellular immortalization and cancer, a large study with 18,430 samples from tumor and normal tissues from 31 different cancer types identified telomere length of the tumor tissue to be shorter than the corresponding normal tissue in majority of the cancer types (18). In line with this, numerous studies on telomere length associations have shown that CLL tumors have significantly shorter telomere length but higher telomerase expression and activity compared to normal B-cells. Thus in cancers, the genomic instability associated with telomere dysfunction may promote selection of fit clones which bypass the senescence checkpoints promoting tumorigenesis while activation of telomerase or ALT serves to maintain the minimal telomere length to overcome senescence and sustain cell survival.

## Methodology for Analysis of Telomere Length in Clinical Samples

Various techniques have been used for the assessment of telomere length in CLL. Telomere length analyzed by telomere restriction fragment (TRF) analysis is considered to be the gold standard. The method includes the process of using a restriction enzyme that does not detect the telomere repeat sequence to digest the non-telomeric DNA, followed by resolution on a gel and southern hybridization (19, 20). Even though the method is highly reproducible, TRF analysis of telomere length has many limitations. Telomere length analyzed using TRF may substantially vary depending on the restriction enzymes used to digest the non-telomeric DNA (21). Additionally, TRF method is not capable of reliably analyzing very short telomeres due to the requirement of hybridization with a probe. The method is low throughput and requires micrograms of DNA. Since the restriction enzymes might not effectively digest the telomere-associated sequences (TAS) that are adjacent to the telomeres, the method usually overestimates the telomere length of a sample (22). Over the years, newer and high-throughput methods for estimation of telomere length were developed, which made analysis of larger patient cohorts easier.

Fluorescence *in situ* hybridization (FISH) using fluorescence labelled (CCCTAA)_n_ telomere binding probes are used for analyzing telomere length, where the intensity of the signal directly corresponds to the length of the telomere sequence in a given sample. The method when coupled with chromosomal banding is a valuable tool for analyzing telomere length of individual chromosomes. FISH based telomere length measurements could be made high-throughput by using flow cytometry (flow-FISH) (23). Another advantage of flow-FISH is that it can be used to analyze telomere lengths of different cell sub-populations within a given sample by using cell-type specific antibodies.

However, the most widely used technique for telomere length measurement is by qPCR, based on a method devised by Cawthon et al. (24). In brief, qPCR technology is used to detect the amount of telomere sequences per sample (T) by using a telomere specific primer and normalizing it with a single copy gene (S) to obtain the average telomere length per cell. The method could be used for relative estimation (T/S ratio) or for absolute telomere length analysis when used with telomere and single copy gene standards (22). The drawbacks of the TRF, flow-FISH, and qPCR based methods is that they provide a mean telomere length of the sample under analysis and not the chromosome specific telomere length. Therefore, to understand telomere length of specific chromosomes with high resolution, the single telomere length amplification (STELA) assay was developed (25). This PCR based method includes ligation of a linker sequence called telorette to the 5’ end of the complementary C-rich strand, followed by amplification of the telomere of a specific strand using telorette and chromosome or allele specific primers. The PCR products are analyzed by Southern blotting or qPCR.

In addition to the above methods that were used for telomere length analysis in CLL, newer techniques have been developed for analyzing different aspects of telomere length. The STELA PCR is capable of analyzing critically short telomeres only on a subset of chromosomes such as XpYp that have unique subtelomeric sequences suitable for designing chromosome specific primers. This limitation was overcome by the universal STELA method (U-STELA) (26). The technique involves digesting the DNA using the enzymes *MseI* and *NdeI* that do not digest the telomeric repeats, followed by ligating adapters complementary to the overhangs created by these enzymes. The non-telomeric parts of the genome that have these adapters on both the ends form a pan-handle like structure due to complementarity between the ends, suppressing PCR amplification. On the other hand, the telomeic sequences have a digested 5’ end and a 3’ G rich overhang that is not processed by the enzymes. Ligation of telorette to the 3’ overhang allows specific amplification of telomeres of all chromosomes. This method is useful for genome-wide analysis of the distribution of critically short telomeres.

The STELA and U-STELA, though highly sensitive, they are biased towards detection of short telomeres (<8kb). The method was further improved and the telomere shortest length assay (TeSLA) was developed (27). In TeSLA, an adapter (TeSLA-T) is first added to the G rich 3’ overhang, followed by the use of restriction enzymes *BfaI, CviAII, MseI, NdeI* to digest the non-telomeric DNA as well as the non-canonical sub-telomeric DNA and to generate 5’ AT and TA overhangs. The 5’ ends of the digested DNA are then dephosphorylated to prevent re-ligation of the ends. Double stranded DNA adapters with phosphorylated 5’ AT and TA overhangs containing C3 spacers are tagged to the digested ends. Telomeres are then amplified using a primer pair specific for the TeSLA-T and 5’ AT/TA adapters. TeSLA allows high resolution analysis of the distribution of <1 to 18 kb long telomeres.

Novel approaches for telomere assessment such as using CRISPR/Cas9 RNA-directed nickase system to specifically label telomeres followed by high throughput imaging using nano channel array have also been developed. This technique permits mapping and analysis of individual telomeres based on subtelomere repeat elements (SRE) and unique sequences in the chromosomes. Recently, another method for telomere length measurement by molecular combing or DNA fiber analysis was reported (28) where cells were embedded in agarose plugs followed by protein digestion to obtain unsheared DNA. The DNA was then solubilized and stretched on cover slips with a constant stretching factor of 2kb/µM. Telomeres were analyzed using a telomere specific PNA probe and the DNA is counterstained to validate the terminal location of the telomeres in the chromosomes. Fluorescence microscopy is used to obtain the distribution of telomere lengths within a sample. The method is reported to be sensitive for estimation of telomere lengths of <1 to >80 kb. In CLL, the dynamics of telomere length distribution in cases with stable and progressive disease is not well defined. The above mentioned novel methods may be valuable in monitoring changes in telomere length landscape within a given case over time and its contribution to clonal diversification, genomic complexity, and disease evolution.

Due to the wide range of methods used for telomere length analysis, the comparability of telomere lengths analyzed in different CLL studies are limited. Moreover, while TRF and STELA based methods have greater reproducibility, qPCR and FISH based methods need to be very carefully and extensively optimized to limit batch effects (29). One of the methods to improve the use of telomere length as comparable biomarker would be to have a standardized set of control samples with telomere length estimated by TRF, included in every batch of FISH or qPCR based analyses to detect and normalize for batch variations and to convert the measured relative (T/S ratios or relative fluorescence units) telomere length as absolute (TRF) values in kilo bases (kb).

## Telomere Length Associations and Prognostic Impact of Telomere Length in Chronic Lymphocytic Leukemia

Early studies on telomere length associations in CLL using TRF analysis of relatively small patient cohorts (n = 58 and n = 61) (30, 31) suggested an association of short telomere length with advanced disease stages, presence of the poor prognostic unmutated IGHV and inferior overall survival (30). Subsequent studies using TRF (31, 32), flow-FISH (33), and q-PCR (34) identified associations of short telomere length with other adverse disease features such as CD38 and ZAP70 expression (35) or lymphocyte doubling time (33). Analysis of telomere length associations with genomic aberration subgroups consistently showed significant association of short telomeres with the poor prognostic, deletion 17p (17p-) and deletion 11q (11q-) while long telomere length was found in cases with deletion 13q (13q-) (36–42). Of note, *TP53* and *ATM* which are critical checkpoint genes activated upon telomere shortening and dysfunction are found in the minimally deleted regions of 17p- and 11q-, respectively. Deletion of these genes therefore permits these tumor cell clones to undergo further telomere shortening compared to non-17p-/11q-, without activating cell death pathways. In line with this, short telomere length was found to be associated with the presence of mutations in *TP53* (37, 40, 41, 43) and *ATM* (41, 43, 44). Cases with 17p- or *TP53* mutation but long telomere length were found to have mutated IGHV (40, 43).

Among the recurrently mutated genes in CLL, *SF3B1* was found to be associated with short telomere length across different studies (37, 40, 43, 45). For *NOTCH1* mutations, some reports suggested an association (37) while others found no association (40, 43) with telomere length. Additionally, Beta-2 microglobulin (ß2M) and serum thymidine kinase (s-TK) levels were also found to be significantly associated with telomere length in CLL. Overall, the presence of short telomere length was found to be significantly associated with various other poor prognostic clinical and genetic characteristics in CLL which translates into an inferior survival compared to those with longer telomere length. Despite this strong association with other disease features, telomere length was found to be an independent prognostic factor in different patient cohorts (35, 36, 39, 40, 42, 43, 46). Accordingly, telomere length was shown to identify poor or favorable risk patients within established prognostic subgroups defined by e.g. IGHV, 17p- and 11q-. Overall, the findings suggest telomere length to be a very important prognostic factor in CLL that could be instrumental for risk stratification as well as monitoring and early detection of changes in clonality. The prognostic impact of telomere length in CLL has so far been established only in chemo or chemo-immunotherapy based trials and it would be interesting to study the telomere length associations in the context of novel therapy.

## Telomere Length and Genomic Complexity

Critical shortening of telomere length, de-protection at telomeres along with loss of checkpoint genes leads to development of genetic lesions and tumorigenesis (16). In CLL, various studies have analyzed the impact of telomere dysfunction on genomic complexity. Early indicators of telomere dysfunction is the formation of DNA damage foci at the telomeres called telomere dysfunction induced foci (TIF) (47). CLL cells were found to exhibit TIF as detected by the localization of gamma H2AX and 53BP1 at the telomeres. In addition, an increase in abnormalities such as telomere deletion/doublets and terminal duplications were observed in TIF+ CLLs (48).

Activation of DNA damage response and DNA repair signaling at the telomeres lead to telomeric fusions. In CLL using STELA method, frequency of telomeric fusion events were found to increase with advancing disease stage and 58% of the Binet C stage had critically eroded telomeres and fusions. Cases having telomeric fusions also showed large scale genomic rearrangements at the telomeric regions (49), reminiscent of genomic complexity due to BFB cycles in telomeres at crisis (16). Subsequently, by analysis of large patient cohort (n = 321), the XpYp telomere length of 2.26 kb was defined as the mean length at which fusions occur (50).

Different studies analyzed the correlation of telomere length with genomic complexity, either by conventional FISH or by SNP array analysis. The analyses showed significant association of short telomeres with presence of two or more aberrations (FISH) (36, 38, 51) or with higher number of copy number alterations (CNAs) (37, 40). Of interest, we observed progressive shortening of telomere length with increase in number of copy number variations (CNVs) (40). Additionally, short telomeres in CLL were also found to be associated with increase in uni-parental disomy (UDP) and chromothripsis (52). The strong association of telomere shortening with terminal fusions and genomic complexity highlights the central role played by telomere dysfunction in clonal diversification and disease evolution in CLL.

## Telomere Length Associations—Cause or Consequence?

**Figure 1:**
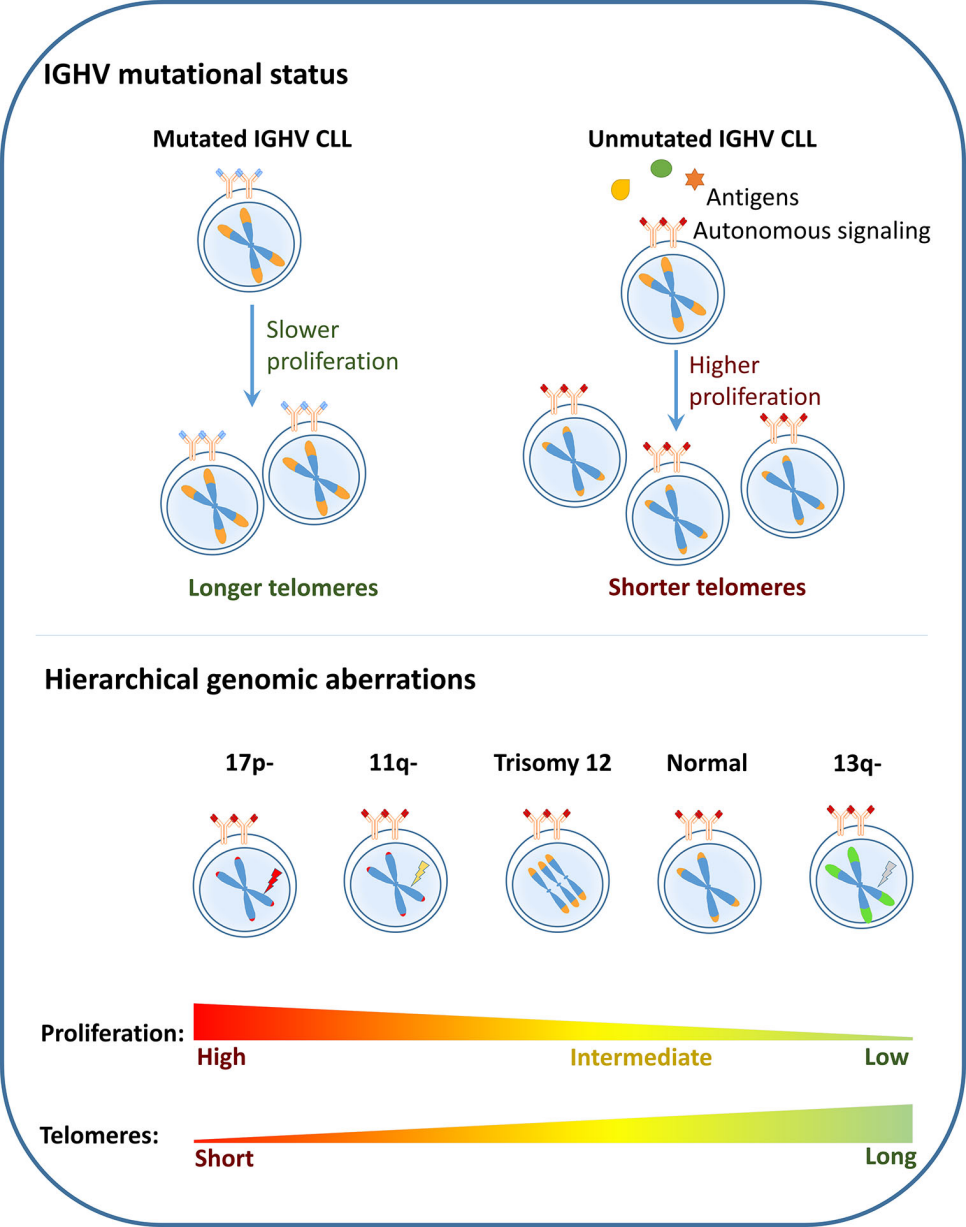
Telomere dysfunction as a consequence: In CLL, the poor risk disease features such as unmutated IGHV, presence of deletion 17p (17p-), deletion 11q (11q-) are shown to be associated with short telomere length while the favorable prognostic subgroups such as mutated IGHV and deletion 13q (13q-) are associated with longer telomeres. It could therefore be considered that telomere length associations are a direct outcome of the proliferation capacity of the different CLL subgroups.

The associations of short telomeres with various adverse prognostic markers such as unmutated IGHV, and *TP53/ATM* mutations, 17p-, 11q- could be explained as a direct outcome or “consequence” of increased proliferation of the cells harboring these high risk features (53). This is supported by the fact that telomere length in serially sampled CLL samples show shortening, despite the presence of active telomerase (33, 40). In addition, Röth et al. identified shorter telomere length of naïve and memory T-cells from patients with more aggressive ZAP-70^+^/CD38^+^ CLL which may be due to increased proliferation and expansion of T-cells in this CLL subtype (54). These findings show that at least in part, the distribution of telomere length among the different CLL subgroups is a direct consequence of their proliferation capacity (**Figure 1**).

On the other hand, telomere length could be considered to play a more active biological role in CLL by being a “cause” for clonal diversification and disease progression. The strong association of telomere length with mutation status of IGHV has been documented across all the studies, owing to differences in the cell of origin. Mutated IGHV CLLs are considered to develop from CD5(+), CD27(+), post-germinal center (GC) B-cell subsets (55), where a robust telomerase activation and elongation of telomere length is known to occur during the GC reaction (56). The non-GC origin of the unmutated IGHV CLL thus may explain the strong association of this subtype with short telomere length.

Telomere shortening has been shown to be a tumor suppressive mechanism, where cells with telomere length shorter than a threshold undergo DNA damage checkpoint activation, stalling further telomere shortening and controlling cell proliferation (17). In CLL cells with unmutated IGHV, the presence of short telomere length may exert a strong selection pressure for loss of checkpoint genes such as *TP53* or *ATM* which would eventually allow for further telomere shortening and cell proliferation. This notion is supported by study on temporal association of genomic alterations in CLL, where 17p-/*TP53* mutations and 11q-/*ATM* mutations were found to be later events in CLL pathogenesis (57). Moreover, we observed in a large clinical trial cohort (n = 620) that cases with 17p- and 11q- had the shortest telomere length across the different genomic aberration subgroups and interestingly, these cases had very short telomeres even when these aberrations were observed in only a small fraction of the tumor bulk. The finding suggested that critical telomere shortening in these cases could precede acquisition of these high-risk aberrations (40). High resolution analysis of genomic fusions in cases with dysfunctional telomeres showed complex inter/intra chromosomal and terminal fusions involving the telomere loci in all of the samples analyzed (n = 9). Strikingly, the telomere fusions also included the loci recurrently altered in CLL (58).

**Figure 2:**
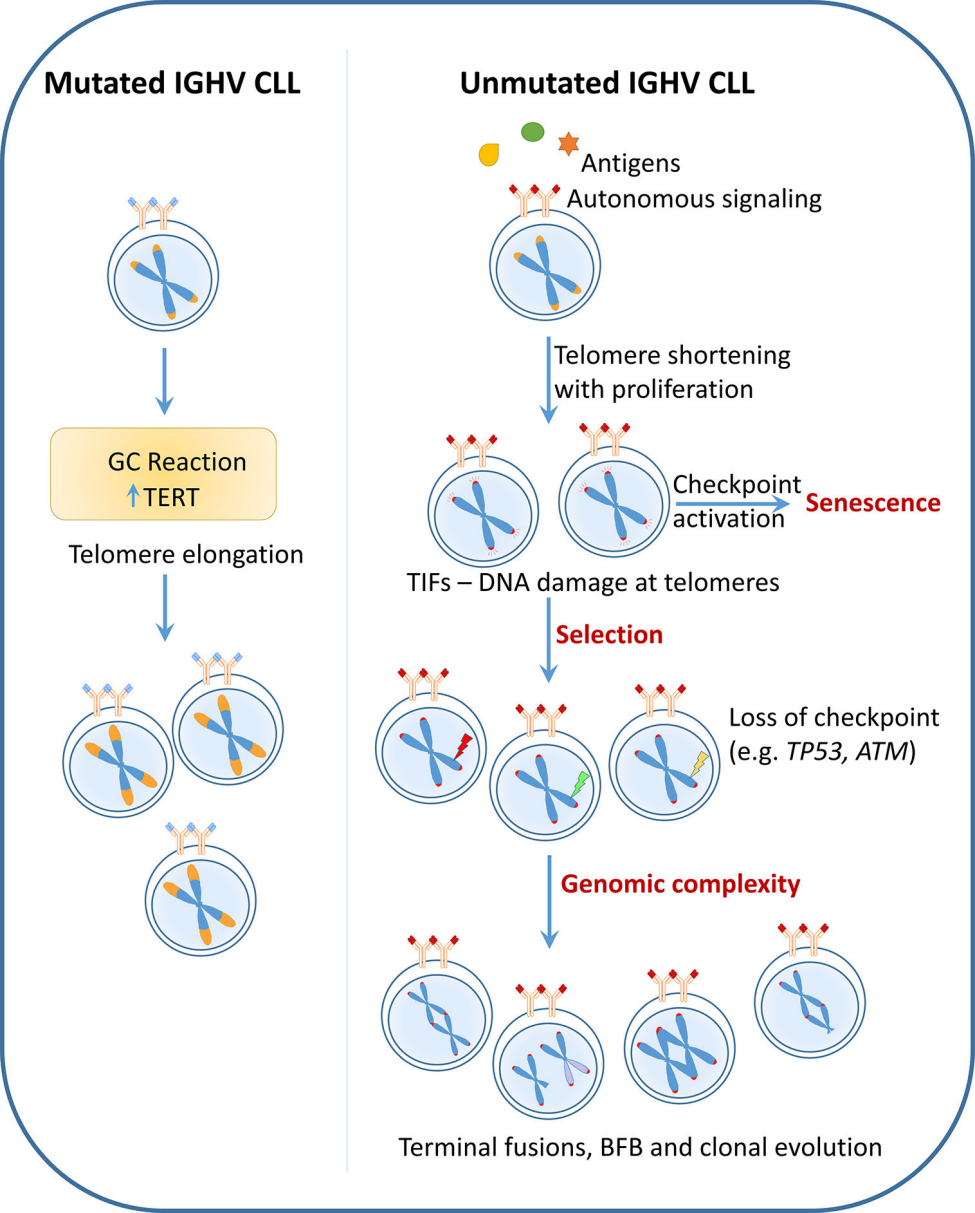
Telomere dysfunction as a cause: CLL with mutated IGHV undergo telomerase activation during the germinal center (GC) reaction leading to telomere elongation. These long telomere length cases follow an indolent disease course and rarely acquire poor-risk features. On the contrary, unmutated IGHV CLL which have poly reactive BCRs undergo progressive telomere shortening with increasing cell proliferation. Critical telomere shortening leads to activation of DNA damage signaling at the telomeres indicated by the presence of telomere dysfunction induced foci (TIF). Persistent DNA damage at the telomeres may lead to selection of clones with dysfunctional checkpoints (e.g. TP53 or ATM loss). The presence of very short telomere length together with absence of checkpoint genes causes telomere fusions, breakage-fusion-bridge (BFB) cycles, eventually leading to heterogeneity and clonal evolution. Thus according to this hypothesis, telomere length which is defined very early in pathogenesis based on cell of origin plays an active role in disease evolution and progression.

Therefore, even though telomere shortening and its association with poor prognostic features could be a consequence or outcome of these poor risk characteristics, recent findings indicate a dynamic role of dysfunctional telomeres in shaping the disease course. Critical telomere shortening confers selection pressure to acquire poor-risk variants and increases disease heterogeneity due to genomic fusion events involving dysfunctional telomeres thereby promoting disease progression and treatment resistance in conjunction with clonal evolution (**Figure 2**).

## Telomerase Expression and its Relation to Disease Features

Activation of the enzyme telomerase is considered as one of the hallmarks of malignant transformation (6) and is pivotal for sustaining cell proliferation. The predominant mechanism of TERT activation in human cancers is by acquisition of *TERT* promoter mutations. In contrast, such mutations are rarely reported in CLL. Ten percent of cancers that do not depend on telomerase depend on ALT mechanism (59). However, a study on the presence of C-Circles and extra chromosomal telomeric repeats (ECTR) which are hallmarks of ALT did not reveal the presence of ALT driven telomere maintenance in CLL (60).

Telomerase activity and/or expression in CLL has been studied across various cohorts. Initially, higher telomerase activity was found to be associated with advanced disease stages and progressive disease (30, 61). Telomerase activity was found to have an inverse correlation with telomere length (33, 62) and higher telomerase expression was associated with other poor-risk disease features and was described as a prognostic factor in CLL (42, 63, 64). Thus, intriguingly, unmutated IGHV CLLs, despite the absence of GC mediated TERT activation and telomere lengthening, these cases have short telomeres but high telomerase expression and activity (65). This indicates that the high TERT expression in unmutated IGHV CLL is therefore crucial for the maintaining the critical telomere length to ensure cell survival and proliferation. However, in contrast to mutated IGHV CLL, processes underlying the high telomerase expression and activity in unmutated IGHV CLL are not well defined.

## Tumor Microenvironment and Telomere Dysfunction

With the absence of the classical oncogenic promoter *TERT* mutations in CLL, the mechanisms underlying its activation are poorly understood. Genome wide association studies repeatedly identified *TERT* as one among the susceptibility loci for risk of CLL (66, 67). Studies to identify SNPs in *TERT* and *TERC* associated with CLL identified the minor rs35033501 *TERT* variant (68), as well as the SNPs rs10936599 in *TERC* and rs2736100 in *TERT* (69) and presence of longer telomere length to be associated with CLL. Though shortening of telomere length in CLL is well characterized to be an adverse prognostic factor it should therefore be noted that telomerase activation and telomere lengthening constitute an important phase in malignant transformation. Also, in cases with poor risk features and rapid disease progression, constant lengthening of telomeres by telomerase is the key to sustain cell survival to counteract telomere loss due to proliferation.

CLLs with unmutated IGHV are known to have a poly-reactive/auto-reactive BCR in contrast to that of mutated IGHV. Apart from this, the CLL BCRs can also signal through cell-autonomous signaling (70, 71). These findings, along with the clinical success of the BCR signaling inhibitors such as ibrutinib and acalabrutinib (72, 73), highlight the importance of BCR signaling for survival and proliferation of CLL cells. BCR along with activation of co-receptors, drives various downstream mechanisms such as activation of PI3K/AKT, NF-kB (74) and MAPK (75) that dictate proliferation, homing and guide interaction with other cells in the microenvironment. Of importance, Damle et al. showed (76) that stimulation of BCR using multivalent BCR ligand, dextran conjugated anti-μ mAb HB57 (HB57-dex) or bivalent F(ab′)_2_ goat anti-μ antibody led to an increase in telomerase activity, predominantly in CLLs with unmutated IGHV. This BCR driven activation of TERT was accompanied by an induction of cell proliferation. They also identified that the TERT activation was mediated by PI3K/AKT signaling, as the use of a PI3K inhibitor abrogated the BCR mediated TERT activation. Another study identified higher *TERT* and *TERC* expression and activity in *SF3B1* mutated CLL, however the underlying mechanism is not well understood (77).

The tumor microenvironment mediated signaling are known to contribute to activation of TERT in different cancers. In breast cancer, STAT3 was found to activate telomerase expression by binding to the TERT promotor (78). In CLL, a constitutive activation of JAK2/STAT3 signaling has been reported (79) and it would therefore be interesting to understand its role in the regulation of TERT in CLL. Another factor that may be of interest for driving TERT activation in CLL is hypoxia. HIF-1α plays an important role in interaction of CLL cells and the microenvironment (80). HIF-1α (81) as well as the levels of hypoxia (82) are known to regulate the expression and activity of telomerase and impact telomere length. Similarly, the Wnt/ß-catenin pathway is a direct regulator of TERT (83) which could be of relevance in the context of CLL. Overall, various pathways that are active in CLL are described to play a role in TERT activation and investigations on the relevance of these mechanisms in regulation of telomerase in CLL may therefore have therapeutic relevance.

## Mutations and Deregulated Expression of Telomere-Related Genes in Chronic Lymphocytic Leukemia

Different components of the telomere system are found to be mutated or deregulated in CLL. Among the recurrently mutated genes, *POT1* mutations have been reported in about 3.5% of the cases. It is the first telomere structural component known to be mutated in human cancers. *POT1* mutations in CLL occur in the OB1 and OB2 domains that alters its binding to the 3’ telomeric tail, leading to de-protection of the ends and genomic instability. In cell line models, loss of POT1 function led to aberrant lengthening of telomeres (84). Thus *POT1* mutations were associated with complex karyotype and are independent prognostic factors for overall survival in CLL (85).

Whole exome sequencing of 66 familial CLLs revealed the presence of germline deactivating *POT1* mutations in four families as well as in the sheltering components adrenocortical dysplasia homolog (ACD, in two families) and telomeric repeat binding factor 2 (TERF2IP, three families) (86). These telomere component mutations are therefore important pre-disposing factors for CLL, highlighting the important role of telomere dysfunction in CLL pathogenesis. In addition, expression analysis of telomere related genes in different CLL cohorts have identified deregulation of various telomere components. One study identified a significant downregulation of Dyskerin, *TRF1, hRAP1, POT1, hEST1A, MRE11, RAD50*, and *KU80* while *TPP1* and *RPA1* were upregulated compared to normal B-cells (87). Another study reported a downregulation of *TIN2* and *ACD* in a subset of CLLs which correlated with increase in TIF, indicating telomere dysfunction (88). Also, downregulation of the telomere components *POT1*, *TIN2*, *TPP1*, and high *TERT* were found to be associated with adverse outcome (89). The shelterin components play a very important role by tightly regulating access of telomerase to the telomeres. Though the mechanisms underlying deregulation of the shelterin components in CLL is unknown, it could be presumed that the downregulation of these genes would promote access of TERT to the telomeres, which would be crucial in maintaining the critical telomere length to sustain cell survival. However, this deregulated expression of the shelterin components also result in uncapping of the ends and increase in DNA damage signaling and DNA repair, leading to fusion and genomic complexity.

## Telomeres and Telomerase Targeted Cancer Therapies

Since telomere maintenance is one of the key features of cancers, the telomere system has been considered an attractive target for cancer therapy. Accordingly, therapeutic agents targeting various components of telomeres and the different maintenance mechanisms have been developed and studied across cancers. One of the first inhibitors of telomerase to have progressed to clinical trials is imetelstat. It is a synthetic lipid conjugated 13-mer oligonucleotide that competitively binds to hTR, thereby inhibiting telomerase function (90). In vitro analysis showed that the drug sensitized primary CLL cells to fludarabine (91). Imetelstat is currently being investigated in phase 2 and 3 trials for various solid tumors and hematological malignancies as a single agent or in combination therapies. Small molecule inhibitors of telomerase such as BIBR1532 are currently under pre-clinical evolution (92). Recently, a covalent telomerase inhibitor (NU-1) that targets the catalytic active site of telomerase has been developed (93). The main disadvantage of telomerase inhibitors is the necessity for continuous long term treatment to impede telomere maintenance and critically shorten the telomere length. Moreover, long term treatment with telomerase inhibitors may additionally affect the function of germ cells and stem cells that express telomerase.

Another class of molecules that affect telomerase activity include nucleoside analogs such as 6-thio-2’-deoxyguanosine (6dG), didanosine (ddITP), azidothymidine (AZT-TP), and 5-fluro-2’deoxyuridine (5-FdU). These compounds when incorporated at the telomeric ends by telomerase leads to chain termination and uncapping of the telomeric ends (94). Uncapping by nucleoside analogs prevents binding of the shelterin complex, thereby activating DDR. Unlike telomerase inhibitors, treatment with nucleoside analogs leads to rapid induction of cell death irrespective of the telomere length. Similarly, compounds such as telomestatin which are G-quadruplex stabilizers lead to impaired telomere maintenance by telomerase thereby inducing DDR and cell death (95, 96).

Though limited clinical progress has been achieved with inhibitors of telomerase, various telomere based immunotherapies are successfully being evaluated in clinical trials for different malignancies. Since telomerase is one of the most commonly expressed tumor associated antigen, different methods are being employed to activate adaptive immune responses against telomerase. TERT peptide vaccines such as INO-1400 (NCT02960594—solid tumors), GV1001 (NCT04032067—Benign Prostatic Hyperplasia), UCPVax (NCT04263051—non-small cell lung cancer), and GX301 (97) are currently being tested in clinical trials for cancer therapy. Of note, DNA vaccine encoding hTERT is being evaluated in a phase 2 study for CLL (NCT03265717). Additionally, adoptive transfer of dendritic cells expressing TERT mRNA (GRNVAC1—NCT00510133) is being studied for the treatment of AML. Another interesting therapeutic approach includes the use of oncolytic adenovirus that replicates under the control of hTERT promoter thereby specifically targeting the tumor cells. The oncolytic adenovirus based therapy telomelysin (OBP-301) is currently being studied for the treatment of a wide range of solid cancers across 6 different clinical trials.

In summary, though the direct inhibition of telomerase has shown limited success, hTERT based immunotherapy are rapidly gaining importance for the treatment of a wide range of tumor entities. In CLL, the novel agents such as ibrutinib and venetoclax have achieved tremendous clinical success however, treatment of Richter transformation has still proved to be challenging. Since Richter syndrome is a highly proliferative tumor type, they might have a greater dependency on telomerase than CLL and hence the novel TERT based immunotherapies either as single agents or in combination with checkpoint inhibitors maybe of interest.

## Conclusion

The relation between telomeres and CLL is complex. Though a large amount of effort has been put forward in understanding the prognostic relevance of telomere length and telomerase, various other aspects such as mechanisms underlying telomerase activation and molecular alterations leading to deregulation of telomere maintenance system still needs to be understood. In summary, deregulations of the different components of the telomere system play important roles at specific phases of CLL pathogenesis and progression. A deeper understanding of these mechanisms is vital for the development of therapeutics options for targeting these disease features, especially in patients that turn refractory to novel agents, or as combination treatments to improve efficacy or in the treatment of Richter transformation.

## Author Contributions

The authors BMCJ and SS wrote the manuscript. Both authors contributed to the article and approved the submitted version.

## Funding

This study is supported by Deutsche Forschungsgemeinschaft (DFG) (SFB 1074 projects B1 and B2).

## Conflict of Interest

The authors declare that the research was conducted in the absence of any commercial or financial relationships that could be construed as a potential conflict of interest.
